# Optimised electroporation mediated DNA vaccination for treatment of prostate cancer

**DOI:** 10.1186/1479-0556-8-1

**Published:** 2010-02-05

**Authors:** Sarfraz Ahmad, Garrett Casey, Paul Sweeney, Mark Tangney, Gerald C O'Sullivan

**Affiliations:** 1 Cork Cancer Research Centre, Mercy University Hospital, Cork, Ireland; 2 Leslie C. Quick Jnr. Laboratory, University College Cork, Cork, Ireland; 3 Department of Urology, Mercy University Hospital, Cork, Ireland

## Abstract

**Background:**

Immunological therapies enhance the ability of the immune system to recognise and destroy cancer cells via selective killing mechanisms. DNA vaccines have potential to activate the immune system against specific antigens, with accompanying potent immunological adjuvant effects from unmethylated CpG motifs as on prokaryotic DNA. We investigated an electroporation driven plasmid DNA vaccination strategy in animal models for treatment of prostate cancer.

**Methods:**

Plasmid expressing human *PSA *gene (phPSA) was delivered *in vivo *by intra-muscular electroporation, to induce effective anti-tumour immune responses against prostate antigen expressing tumours. Groups of male C57 BL/6 mice received intra-muscular injections of phPSA plasmid. For phPSA delivery, quadriceps muscle was injected with 50 μg plasmid. After 80 seconds, square-wave pulses were administered in sequence using a custom designed pulse generator and acustom-designed applicator with 2 needles placed through the skin central to the muscle. To determine an optimum treatment regimen, three different vaccination schedules were investigated. In a separate experiment, the immune potential of the phPSA vaccine was further enhanced with co- administration of synthetic CpG rich oligonucleotides. One week after last vaccination, the mice were challenged subcutaneously with TRAMPC1/hPSA (prostate cancer cell line stably expressing human *PSA*) and tumour growth was monitored. Serum from animals was examined by ELISA for anti-hPSA antibodies and for IFNγ. Histological assessment of the tumours was also carried out. *In vivo *and *in vitro *cytotoxicity assays were performed with splenocytes from treated mice.

**Results:**

The phPSA vaccine therapy significantly delayed the appearance of tumours and resulted in prolonged survival of the animals. Four-dose vaccination regimen provided optimal immunological effects. Co - administration of the synthetic CpG with phPSA increased anti-tumour responses, preventing tumour occurrence in 54% of treated animals. Vaccination with phPSA resulted in anti-hPSA Abs production and a significant production of IFNγ was observed in immunised animals (p < 0.05). Immune responses were tumour specific and were transferable in adoptive T cell transfer experiments.

**Conclusions:**

This phPSA plasmid electroporation vaccination strategy can effectively activate tumour specific immune responses. Optimisation of the approach indicated that a four-dose regimen provided highest tumour protection. *In vivo *electroporation mediated vaccination is a safe and effective modality for the treatment of prostate cancer and has a potential to be used as a neo-adjuvant or adjuvant therapy.

## Background

Prostate cancer remains a major health issue in the present era, largely due to limitation of therapeutic options especially in advanced disease. Prostate cancer represents the most common non-cutaneous cancer and is the second leading cause of cancer related deaths among American men [1]. There are continuing efforts to discover new treatments for prostate cancer, in particular for advanced disease. Novel therapeutic strategies are needed to prevent progression from localised to advanced disease and to further improve survival outcomes in patients with metastatic disease. Manipulation of the immune system and destruction of cancer cells by the immune activated mechanisms have shown promising results in the treatment of malignant diseases [2].

Healthy individuals are known to have some immune inhibitory effects on prostate cancer growth (at least early phase of the disease), while progressive tumour development is a result of tumour escape from the immune system. Many factors are involved in tumour immune escape. Blades et *al*. [3] have shown the reduction of MHC-1 expression in 34% of primary prostate cancer and 80% tumours associated with lymph node metastases. Other causes include secretion of inhibitory substances e.g. IL-10, TGF-β [4], abnormal T-lymphocyte signal transduction [5] and expression of Fas ligand, which may enable tumour cells to induce apoptosis in Fas expressing tumour infiltrating lymphocytes [6]. Immunological therapies may overcome these escape pathways and can potentially play an effective role in the management of prostate cancer in isolation or in conjunction with available therapies. Patients with advanced prostate cancer are known to have defective cell mediated immunity [7] . Both antibody and CD8^+ ^T-cell immune responses have been reported in patients with advanced prostate cancer [8-10] .

For malignant diseases different approaches of active immunisation have been explored, including vaccination with cDNA [11] , RNA [12], proteins or peptides [13]. Over the past years, several prostate cancer associated antigens have been reported including prostate specific antigen (PSA), prostate-specific membrane antigen (PSMA) [14], prostate stem cell antigen (PSCA) [15] and six transmembrane epithelial antigen (STEAP) [16]. We have previously demonstrated the potential for electroporation (EP) mediated DNA vaccination with PSCA [17]. In the present study, we focus on optimisation of *in vivo *DNA plasmid vaccination, in terms of dose schedule and combination with CpG oligonucleotides. We investigated the utilisation of a human PSA expressing plasmid in a murine model of prostate cancer. PSA, a serine protease secreted by both normal and transformed epithelial cells, is almost exclusively expressed on prostatic epithelial cells, and its expression is conserved in nearly all advanced prostate cancer [18]. PSA is widely used as marker for diagnosis and staging of prostate cancer [19]. Although PSA is a secreted protease, MHC related epitope processing in target PSA expressing cells has been shown to make PSA a valid target for vaccination [20]. Additionally, a DNA vaccination with plasmid encoding PSA has a potential to evoke specific anti-tumour cellular immune responses [21].

DNA vaccines induce immune responses by direct expression of the antigen by the host cells. Electric pulse parameters optimal for the plasmid delivery have been shown to enhance humoral immune responses [22]. Moreover, plasmid DNA contains CpG motifs, which are immune-stimulatory and have been shown to induce potent immunological adjuvant effects [23,24]. While gene based vaccines for prostate cancer have been studied previously, optimal vaccine schedule with EP driven plasmid delivery has not been evaluated. This study aims to test various EP vaccination regimens for prostate cancer in an animal model.

## Methods

### Plasmids

The human PSA (*hPSA*) expressing plasmid pUMFG/PSA/IRES/CD25 (hereafter referred to as phPSA) was kindly supplied by Jeffrey A Medin, Division of Experimental Therapeutics, Ontario Cancer Institute, Toronto, Canada [25]. Vaccine gene free backbone plasmid (empty vector) was generated in our laboratory for use in control groups. For *in vivo *vaccination, plasmid DNA was prepared using an Endotoxin free mega kit (Qiagen, West Sussex, UK). Required plasmids were confirmed by enzyme digest and running on 1% agarose gel (Sigma, Dublin, Ireland). Group of mice were also treated with firefly luciferase plasmid (pCMV- luc). The firefly luciferase gene under the control of the CMV promoter was provided by Plasmid Factory GmbH (Bielefeld, Germany). Plasmid concentrations were determined with the aid of the Nano Drop 1000 Spectrophotometer (Thermo Scientific, MA, USA).

### Cell lines

The murine recycled prostate cancer cell line TRAMPC1 was kindly provided by RP Ciavarra [26] of Eastern Virginia Medical School, Norfolk USA. TRAMPC1 cells were stably transfected with phPSA, using Fugene (Roche, West Sussex, UK) according to manufacturer's instructions. The *hPSA *expressing stable transfected clone was generated following selection with 200 μg/ml of Geneticin (Invivogen, Cayla, France). The clone was then isolated and purified following three rounds of single cell dilution and designated TRAMPC1/hPSA. The expression of *hPSA *in TRAMPC1/hPSA was analysed by isolation of RNA and reverse transcription polymerase chain reaction (RT-PCR). For RT-PCR, first strand cDNA was synthesised using omniscript reverse transcription kit (Qiagen, West Sussex, UK). The *hPSA *cDNA was amplified by PCR, using *Pwo *polymerase (Roche, West Sussex, UK), with *hPSA *foreword primer (5'-GCAGCATTGA ACCAGAGGAG-3') and *hPSA *reverse primer (5'-CGATGGTGTC CTTGATCCAC-3'). PCR reaction conditions included 15 min of initial denaturation at 95°C followed by 32 cycles of 1 min at 94°C, 1 min at 57°C, 1 min at 72°C. The wild-type and transfected TRAMPC1 cells were grown in culture at 37°C as reported previously [17].

### Animals and tumour induction

Male C57 BL/6 or MF1-nu/nu mice, 6 - 8 weeks old were used in the study. The mice were obtained from Harlan Laboratories (Oxfordshire, England). The animal ethics committee of University College Cork approved all experiments. Mice were kept at a constant room temperature (22°C) with a natural day/night light cycle in a conventional animal colony. Standard laboratory food and water were provided. All mice were maintained in a pathogen free animal facility for at least 2 weeks before the experiments. Subcutaneous (s.c.) tumour inoculation and the tumour growth measurements were recorded (on average every 2 days) as reported previously [17]. A mouse was considered incurable and euthanised by cervical dislocation when the tumour diameter reached 1.5 cm. From these volumes tumour growth curves were constructed. All of the immunological data reported is representative of at least two independent studies. Each study was performed with 5 or 6 mice per group.

### Optimisation of *in vivo *vaccination

**Figure 1:**
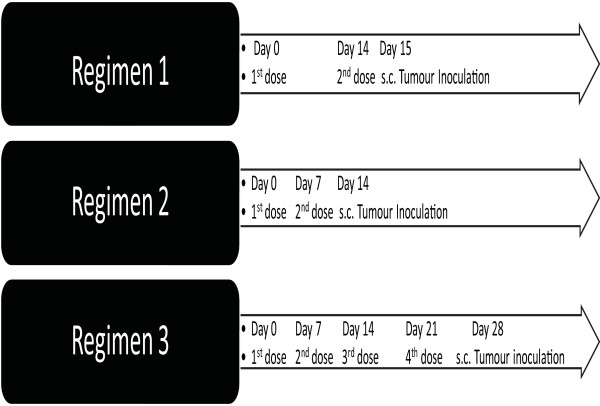
**Schematic representation of vaccination schedules**. Regimen1 involved two vaccinations (day 0 and 14) with subsequent tumour challenge on day 15. Regimen 2 involved vaccinations on day 0, 7, and tumour challenge on day 14. In regimen 3, four doses of vaccine were given on day 0, 7, 14, and 21 followed by tumour challenge on day 28.

Male C57 BL/6 mice were randomly divided into three groups; phPSA, empty vector and untreated. Mice were anaesthetised during all treatments by intra-peritoneal (i.p.) administration of 200 μg xylazine and 2 mg ketamine. For vaccine delivery, a custom-designed applicator (Cliniporator, IGEA, Modena Italy) with two needle electrode (4 mm apart) was used. Both needles were placed through the skin central to the quadriceps muscle. The muscle was injected between electrode needles with 50 μg plasmid DNA in 50 μl sterile phosphate buffer saline (PBS). After 80 s, square-wave pulses (1200 V/cm 100 ms × 1 and 120 V/cm 20 ms, eight pulses) were administered in sequence using a custom-designed pulse generator (Cliniporator). The untreated group did not receive EP. To determine the optimum vaccination protocol, three different regimens of vaccination were tested (Figure 1).

### Confirmation of antigen expression in muscle tissues

The ability to deliver plasmid DNA into the quadriceps muscles, using this electroporation technique, was demonstrated by electroporation of pCMV-luc and detection of subsequent firefly luciferase expression. The pCMV-luc was injected i.m. [50] μl (30 mg/ml)] followed by EP. After 72 h, firefly luciferin (80 l of 30 g/l conc.) (Biosynth, Basel, Switzerland) was injected intra-peritoneal as a substrate for the luciferase enzyme. Mice were anesthetised as previously outlined. Ten minutes post luciferin injection, live anesthetised mice were imaged for 1 min using an intensified CCD camera (IVIS Imaging System, Xenogen, Caliper Life Sciences, Runcorn, England). After imaging, the mice were culled and the transfected leg was separated and imaged immediately.

Specific gene expression (*hPSA*) by the muscles cells post EP mediated vaccination was demonstrated by RT-PCR of the transfected muscles. After 72 hours of the vaccine delivery, mice were culled by cervical dislocation and quadriceps muscle excised. The muscle tissues were homogenised in TRI Reagent^® ^(Molecular Research Centre, Inc.) to isolate total RNA. DNase treated total RNA from the transfected muscle was subjected to RT-PCR amplification with *hPSA *specific primers at same conditions (as described earlier).

### Antigen specificity and long-term tumour protection

Groups of the regimen 3 treated and untreated mice were challenged either with wild TRAMPC1 or with TRAMPC1/hPSA. All mice developed tumours, these tumours were surgically excised when tumours approached 5-7 mm in major dimension. The animals were observed for another 30 days without recurrence of the tumours. Tumour free mice at that stage were re-challenged, in the opposite flank, with the same tumourogenic dose of the initially challenged tumours (wild or transfected). Any re-growth of the tumours was observed and growth kinetics recorded.

### ELISA

The phPSA induced activation of the immune system and production of interferon gamma (IFNγ), a prototype Th1 cytokine, was tested in vaccinated and naive mice as previously reported [17]. An indirect ELISA was performed for detection of anti-human PSA antibodies. Serum from mice vaccinated with phPSA was analysed for anti-human PSA antibodies. Blood samples from the jaw veins were collected in heparin containing vials from both phPSA treated and from naive mice. To separate plasma the samples were centrifuge for 10 min at 1000 × g within 30 min of collection. Assays were performed either immediately or sample were stored at -20°C for later use. A single sample was tested from each animal at each time point. Blood samples were collected at week 2, 4, 8, and 12 after last vaccination. Plasma from the naive male C57 BL/6 mice was used as control. The 96-well plates were coated with 1 mg/ml of human PSA antigen (Europa Bioproducts, Cambridge, UK) in PBS containing 0.05% NaN3 (PBSN) and incubated at room temperature (RT) overnight. Plates were blocked for 1 hour at 37°C by the addition of 10% rabbit serum diluted in PBSN. After washing three times with PBSN, either PSA mAb (10-P20A, 1 mg/ml) (Europa Bioproducts) in blocking buffer (0.05% Tween 20 and 0.25% BSA in PBSN), as a standard, or 50 μl of mouse plasma samples in blocking buffer, as tests (1:10,1:100, 1:1000 dilutions were used) were added to the plates and incubated overnight at RT. The plates were then blocked again by 1 hour incubation at 37°C in 10% sheep serum, and further incubated with goat anti-mouse IgG conjugated to alkaline phosphatase (Sigma) for 5 hours at RT. After incubation with substrate, (pNPP) qualitative hydrolysis of NPP was detected using a microtiter plate reader (Vmax, Molecular Devices) with a 405-nm filter. Dilutions were also made using blocking buffer to re-assay samples that were beyond linearity for the initial 1000 × dilution.

### *In vitro *and *in vivo *cytotoxicity assay

For *in vitro *cytotoxicity assays, splenocytes were isolated from the phPSA treated and naive mice. CTL activity against the TRAMPC1/hPSA cells was analysed using same protocols reported previously [17,27]. Results of representative experiments are given as the mean +/- standard deviation and of multiple experiments as the mean +/- standard error. The development of an immune mediated anti-tumour activity following treatment was also tested by a modified Winn assay [27,28]. Splenocytes were isolated from the immunised and from the naive mice. Groups of male C57 BL/6 mice received s.c. injections of a mixture of splenocytes (from either vaccinated or naive mice) and the TRAMPC1/hPSA. For the s.c. inoculation, the splenocytes were mixed with TRAMPC1/hPSA (5 × 10^6^) in a proportion of 50:1 in serum- free Dulbecco's Modified Eagle's Medium (Gibco, Paisley, Scotland). Tumour devel-opment after inoculation was monitored on alternate days.

### Co-administration of phPSA with synthetic CpG oligodeoxynucleotides

CpG oligodeoxynucleotides 1826, chosen according to published data [29,30], had the following sequence TTCATGACGTTCCTGACGTT (CpG motifs are under-lined) with the backbone phosphorothioate stabilised. The oligo CpG was synthesised by MWG (Munich, Germany), reconstituted in sterile pyrogen free water, and diluted in PBS for *in vivo *injection. Three days after each application of EP driven vaccination (regimen 3), mice were injected at same site with synthetic oligo CpG (25 μg/injection). Mice in the control groups were injected either with DNA vaccine or with oligo CpG.

### Statistical analysis

The primary outcome variable of the statistical analyses was the tumour volume in each mouse measured at each time point. The principal explanatory variables were the different treatment groups. Tumour volume was analysed as continuous. Treatment groups were analysed as categorical variables. At each time point, a two-sampled t-test was used to compare mean tumour volume within each treatment group. One-way ANOVA was used to compare mean time of tumour appearance in various groups. Animal survival was represented by Kaplan Meyer survival curves. A p value < 0.05 was interpreted as a significant difference. Microsoft Excel 10.0 (Microsoft) was used to manage and analyse data.

## Results

### Immune potential of TRAMPC1

To establish the growth and effects of the naive immune reactivity against the recycled TRAMPC1 cells, s.c. tumour inoculation of the TRAMPC1 was performed in both immunocompetent (C57 BL/6) and athymic nude (MF1-nu/nu) male mice with same tumourigenic dose (5 × 10^6^/mouse). Nude mice developed tumours earlier than C57 BL/6, which grew more rapidly, resulting in decreased survival of the nude mice (data not shown). These results suggested that the presence of intact immunity in C57 BL/6 mice has some inhibitory effects on the growth of TRAMPC1 tumours, indicating that the TRAMPC1 tumour can be targeted using immune base therapies.

*In vivo *growth of the wild TRAMPC1 and TRAMPC1/hPSA tumours in C57 BL/6 was comparable (data not shown). This showed that the presence of the human antigen (*hPSA*) in the TRAMPC1 did not cause significant effects on *in vivo *tumour growth, validating the suitability of the TRAMPC1/hPSA model.

### *In vivo *gene delivery

**Figure 2:**
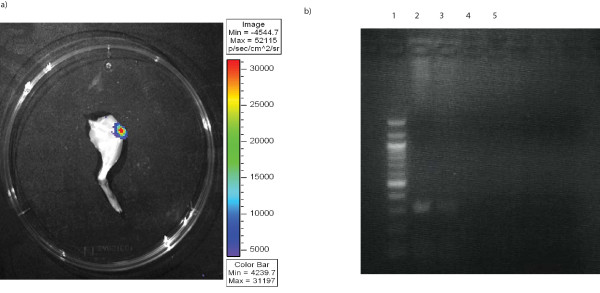
**Electroporation mediate plasmid transfection of quadriceps**. a) *In vivo *muscle transfection by EP was assessed by luciferase activity in resected leg 72 h post transfection (imaged for 1 min using an intensified CCD camera (IVIS Imaging System, Xenogen). b) RT-PCR analysis of mRNA expression of *hPSA *in muscle. *hPSA *was only detected in muscles electroporated with phPSA (lane 1 100 bp marker, lane 2 phPSA transfected muscle (sample a), lane 3 phPSA transfected muscle (sample b), lane 4 empty vector transfected muscle, lane 5 untreated muscle).

*In vivo *luciferase activity was demonstrated after EP mediated transfection. The successful transfection of the quadriceps muscle group is shown in a representative image (Figure 2a). RT-PCR analysis of the muscles treated with phPSA confirmed the successful gene expression in treated mice (Figure 2b).

### Tumour protection by phPSA vaccination

**Figure 3:**
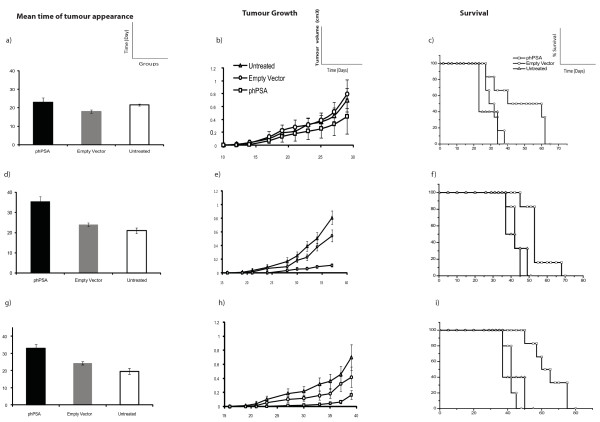
**Tumour protective effects of the various vaccination regimens**. (n = 6) Regimen 1 - a) Time of tumour appearance - the mean time of tumour appearance was comparable in various groups (p = 0.07). b) Representative tumour growth curve - phPSA immunised mice had low tumour volumes but the difference was not significant (p vs empty vector = 0.34, vs untreated = 0.27). c) Representative Kaplan Meyer survival curve - mean survival in the immunised group was significantly prolonged (p vs empty vector = 0.04, vs untreated = 0.03). Regimen 2 - d) Time of tumour appearance - the phPSA treated mice remained tumours free for prolonged period of time (p < 0.01). e) Representative tumour growth curve - tumour growth was retarded in the immunised group. The tumour volumes were significantly lower than the untreated group at all time points (p = 0.04), but not when compared with empty vector group (p = 0.07). f) Representative Kaplan Meyer survival curve - immunisation with phPSA provided significant prolonged survival of the treated mice (p vs empty vector = 0.01, vs untreated = 0.01). Regimen 3 - g) Time of tumour appearance - mean time of tumour appearance was delayed significantly as compared to both control groups (p < 0.01). h) Representative tumour growth curve - tumour growth was significantly retarded in phPSA immunised group (p vs empty vector = 0.04, vs untreated = 0.01). i) Representative Kaplan Meyer survival curve - average survival in immunise group was significantly prolonged (p vs empty vector < 0.01, vs untreated < 0.01). Data are expressed as means ± SEM.

Three different vaccination protocols were examined (Figure 1) using the same EP parameters. All vaccination regimens had variable degrees of inhibitory effects on the tumour growth and animal survival (Figure 3). With regimen 1, mean time of tumour appearance in phPSA group was prolonged, although without statistical significance. The mean time of the tumour appearance was 23 days in hPSA group, 18 days in empty vector and 21 days in untreated group (p = 0.07). However, the rate of tumour growth was lower in the vaccinated group, which resulted in significantly prolonged survival of the phPSA immunised mice. The mean survival in phPSA group was 53 days, 32 days in empty vector and 30.5 days in untreated group (p vs empty vector = 0.04, vs untreated = 0.031) (Figure 3a).

With regimen 2, the mean time of tumour appearance in the phPSA immunised group was 35.5 days, significantly prolonged as compared to the both control groups (p < 0.01). Additionally the tumour growth was much slower in the immunised group than in untreated (p = 0.02). Although the immunisation resulted in slower tumour growth (compared to both control groups), the difference in growth rate between phPSA and empty vector group was not statistically significant (p = 0.06). The mean survival in immunised mice was 55.6 days, 45 days in empty vector and 42.5 days in untreated mice (p < 0.05) (Figure 3b).

**Figure 4:**
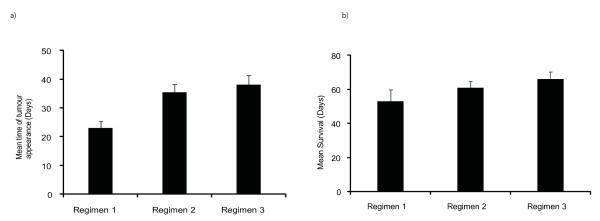
**Comparison of vaccination regimens**. a) Time of tumour appearance in various vaccinated groups. The regimen 2 and 3 resulted in prolonged tumour free periods (p regimen 1 vs 2 < 0.01, regimen 1 vs 3 < 0.01, and regimen 2 vs 3 = 0.4). b) Mean survival in various vaccination regimens (p regimen 1 vs 2 = 0.4, regimen1 vs 3 = 0.04, regimen 2 vs 3 = 0.04). Data shown only for the phPSA vaccinated mice in all three regimens. Errors bars represent SE.

Regimen 3 was found to be the most effective strategy resulting in delay in time of tumour appearance, retarded tumour growth, and prolonged survival of the tumour bearing mice. The mean time of the tumour appearance was 32.8 days in immunised group (p < 0.01). The tumour growth was also much slower as compared to both groups (p vs empty vector = 0.04, vs untreated = 0.01). These effects were translated into prolonged survival with mean survival after tumour inoculation in phPSA immunised group was 67.5 days, 45 days in empty vector and 40 days in untreated group (p < 0.01) (Figure 3c). An overall comparison of the immunised mice in all three regimens is shown in Figure 4. These data indicate the superior immunological and tumour inhibitory effects of the four-dose vaccination. No adverse effects related with repeated vaccination were observed. There were no immunisation related deaths and all mice remained healthy throughout the experimental period.

### Activation of humoral immunity

**Figure 5:**
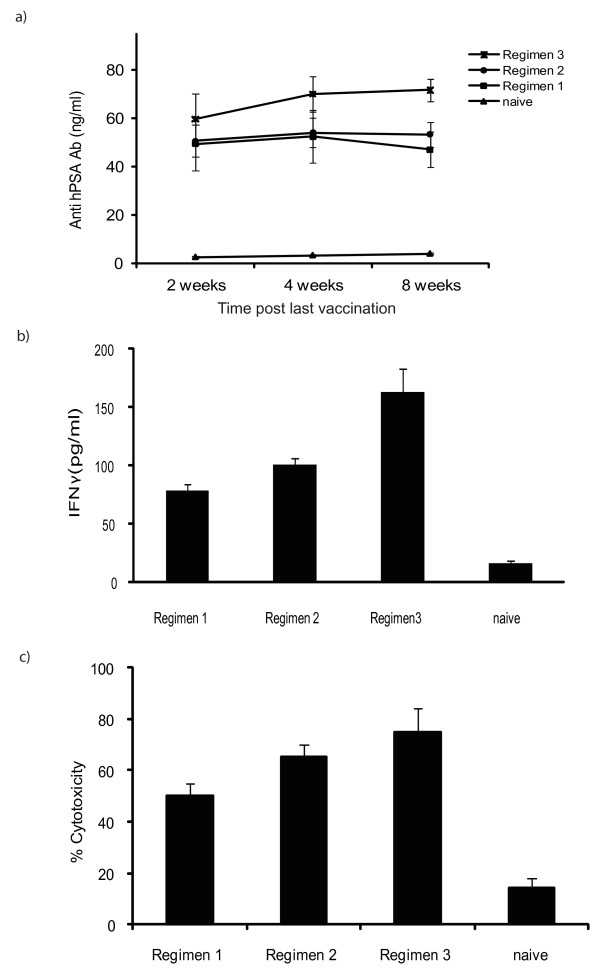
**Induction of protective anti-tumour immunity**. a) Levels of anti-hPSA antibodies at various time points from both vaccinated and from naive mice. Elevated levels of the anti hPSA antibodies were found in the serum from the immunised mice. Regimen 3 resulted in higher levels at all time points. Errors bars represent SE. b) IFNγ production - at study end point in various groups (n = 6), supernatants from the stimulated (48 hours) splenocytes collected and tested for the production of IFNγ. Higher levels of IFNγ were detected in all groups as compared to naive, while regimen 3 resulted in a much higher IFNγ production (p vs regimen 1 < 0.01, vs regimen 2 = 0.01). Regimen 2 levels were also significantly higher as compared to regimen 1 (p = 0.03). c) *In vitro *augmentation of the cytolytic activities of the splenocytes after immunisation - specific cytotoxicity was greatest at an effector target ratio of 20:1 in all vaccination schedules. Maximum cytotoxicity was observed in regimen 3. The % cytotoxicity was significantly higher than naive for all regimens, but differences between regimens were not statistically different (p > 0.05) (p vs regimen 3 = 0.02, vs regimen 2 = 0.01, vs regimen 1 = 0.03). Errors bars represent SE.

Production of anti hPSA antibodies in mice serum was determined at various time points after last vaccination. Higher levels of anti-hPSA antibodies were observed at all study time points in regimen 3 and these levels remained persistently higher for up to 12 weeks after last vaccination. Regimen 1 also resulted in production of the anti-hPSA antibodies, but a drop in the level was observed after 4 weeks (Figure 5a). After 8 weeks from the last vaccination, the assessment of antibodies was not possible in regimen 1 and 2 - as the mice were developing growing tumours and the tumour volumes required culling of the animals to comply with ethical committee guidelines. However in the regimen 3, the mean level of the anti hPSA antibodies was 67.83 at week 12 post last vaccination, indicating that there is persistent production of the antibodies. This factor may be responsible for the superior effects of the regimen 3. At week 2 and week 4 post final vaccinations, the levels of anti hPSA antibodies were not statistically different between the tested regimens (p > 0.05). However, at week 8 significant higher levels of the anti hPSA antibodies were recorded in the mice treated with regimen 3 (p vs regimen 1 = 0.01, vs regimen 2 = 0.02).

### Activation of cell mediated immunity

Histological analysis (H & E) of the tumours from the immunised mice showed abundant lymphocyte infiltration (data not shown). Immunisation with phPSA also resulted in higher production of IFNγ, indicative of Th1 immune activation. On comparison of the different vaccination protocols, variable amounts of the IFNγ was recorded in the treated mice. However, the levels in regimen 3 were much higher than in other protocols. Mean value of the IFNγ in the regimen 3 was 163.3 pg/ml, while 100.33 pg/ml and 78.33 pg/ml in the regimen 2 and 1 respectively (Figure 5b). The regimen 3 was superior to the others (p vs regimen 1 < 0.01, vs regimen 2 = 0.01). Regimen 2 also resulted in more IFNγ activity than regimen 1 (p = 0.03). These observations could explain the better tumour protective effects with regimen 2 and 3 than with regimen 1.

**Figure 6:**
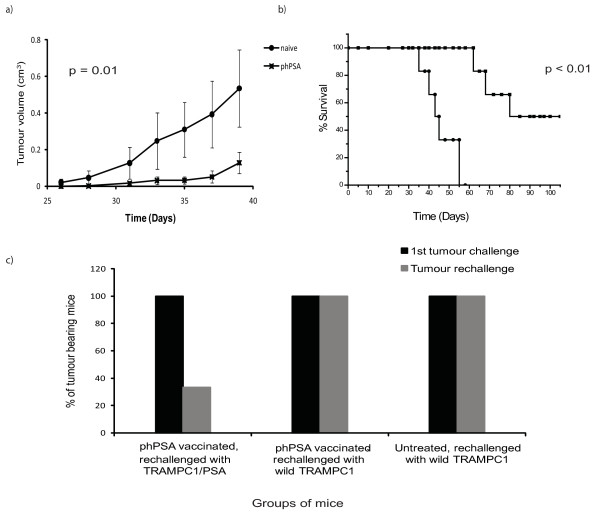
***In vivo *adoptive transfer of lymphocytes and antigen specific response**. a) Low tumour volumes were observed in mice (n = 6) receiving splenocytes from immunised group and this slow growth of the tumours provided a prolonged survival. b). Data are expressed as means ± SEM. c) Antigen specific responses - groups of mice (n = 6) were immunised (regimen 3) and challenged s.c. (1^st ^tumour challenge) either with wild TRAMPC1 or transfected TRAMPC1 (TRAMPC1/hPSA). After surgical excision of the tumours and 30 days period of observation, mice were rechallenged (Tumour rechallenge), either with wild type TRAMPC1 or TRAMPC1/hPSA cells. The vaccine response was antigen specific, as on re-challenge tumour protection was only observed in mice with neo-adjuvant immunisation and rechallenge with TRAMPC1/hPSA. Only 33% mice developed tumour after rechallenge, while remaining mice remained tumour free for more than 100 days post rechallenge with TRAMPC1/hPSA.

*In vitro *cytotoxicity was determined in stimulated splenocytes in all three regimens. Regimen 3 resulted in higher cytotoxicity (75%) than regimen 2 (60%) and regimen 1 (50%) (Figure 5c). *In vivo *cytotoxicity was also demonstrated by modified Winn assay. Splenocytes from vaccinated mice (regimen 3) with longest survival were harvested, mixed with TRAMPC1/hPSA and subsequently inoculated s.c. in groups of C57 BL/6 mice. Fifty percent of the mice receiving mixture of splenocytes from phPSA immunised group failed to develop tumours and importantly the tumour growth in tumour developing mice was significantly retarded (p < 0.01) (Figure 6a). These effects resulted in overall improvement in survival of these mice (Figure 6b).

### *hPSA*-encoding plasmid provided antigen specific protection

After tumour rechallenge with TRAMPC1/hPSA, only 33% mice developed tumours with previous neo-adjuvant phPSA vaccination (Figure 6c). Additionally, the tumour protective effects were specific, as there was no tumour protection following rechallenge with wild TRAMPC1. This demonstrates that phPSA treatment induced a hPSA antigen-specific immune response giving resistance to the same tumour cell line, but not to a wild type (-ve for *hPSA*).

### Co-administration of synthetic Oligo CpG with phPSA

**Figure 7:**
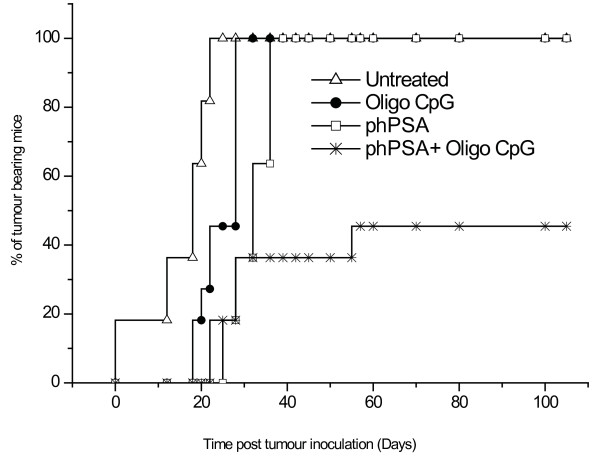
**Time of tumour development**. Co-administration of the synthetic oligo CpG with phPSA (regimen 3) resulted in delayed onset of tumours (n = 11). 54% mice (6 out of 11) remained tumour free for > 100 days post s.c. tumour inoculation. The tumour development in remaining five mice was delayed as compared to other groups.

Use of a synthetic oligo CpG was examined aimed at promoting Th1 type immune responses, to augment potency of the phPSA vaccine. After four vaccines and adjuvant doses of the oligo CpG, 6 out of 11 mice remained tumour free for more than 100 days (cured). Hence, the adjuvant effects of the synthetic oligo CpG resulted in the complete tumour protection (relative risk reduction of 0.45%) as compared with single therapy alone. Time of tumour appearance was also prolonged in combined therapy groups (Figure 7). No significant increase in the levels of anti hPSA antibodies was observed in phPSA + Oligo CpG groups as compared to phPSA alone (data not shown).

## Discussion

Electroporation driven immunisation with prostate antigen (hPSA) encoding plasmid resulted in specific broad immune responses with effective tumour containment. For a cancer vaccine, the prevention of tumour progression may be dependent on both humoral and cellular immunity. We have shown that EP mediated DNA delivery is capable of stimulating both arms of the immune system. Both humoral and cell mediated immune responses were observed as indicated by the anti hPSA antibodies production and *in vitro*/*in vivo *cytotoxicity respectively. These immune responses were antigen specific as in our tumour rechallenge experiments, tumour protection was only observed in mice challenged with transfected cells. In this study, all three tested regimens provided variable effects on tumour growth but repeated vaccination four times on weekly interval resulted more effective immune responses. All mice developed tumours indicating that these regimens did not provide complete tumour protection. Nevertheless, low tumour burden and prolonged survival in immunised mice was achieved with phPSA vaccination with all vaccination regimens. Furthermore, on co-administration of an immune adjuvant, synthetic CpG containing oligonnucleotides, complete tumour protection was achieved in 54% of animals. Immune effects of CpG DNA in infection have been well documented. It has been observed that the release of unmethylated CpG DNA (which is unique to prokaryotes) during an infection provides a 'danger signal' to the innate immune system, triggering a protective immune response that improves the ability of the host to eliminate infecting microbes [31]. This initiates a cascade of events that culminates in the indirect maturation, differentiation, and proliferation of T cells and natural killer cells [32]. Together, these cells secrete cytokines and chemokines that create a pro-inflammatory (IL1, IL 6, IL18 and TNFα) and Th1-polarised (IFNγ, and IL 12) immune environment [33]. These events further facilitate the development of antigen-specific CTLs [34,35]. The induction of these immune responses by oligo CpG has encouraged the idea of a potential role of oligo CpG as vaccine adjuvant. In this study, the CpG adjuvant potentiated the specific anti- tumour immunity as observed by complete tumour growth inhibition.

Developments in tumour vaccines are influenced by the substantial success of the various types of vaccines for infectious diseases. The majority of these vaccines for infectious diseases have effective prophylactic roles with limited utility in therapeutic settings. Tumour vaccine studies have clearly shown that vaccines elicit effective responses against early, microscopic tumours, but are ineffective against established, large tumour masses [36,37]. These observations led to the idea of generation of prophylactic, rather than therapeutic, cancer vaccines [36]. DNA vaccines are simple vehicles for *in vivo *transfection and antigen production leading to induction of immunity. A DNA vaccine can activate the innate immune responses by the presence of hypomethylated CpG dinucleotide sequences with particular surrounding motifs in the bacterial plasmid backbone [38]. This may be a natural response to exposure to a bacterial DNA and is a significant operational component of DNA vaccines. However, this does not completely explain how plasmid DNA is perceived by the innate immune response. Oligonucleotides are known to require Toll-like receptor 9 (TLR-9) for immune-influencing activity, but DNA vaccines operate normally in TLR-9 -/- mice, indicating the involvement of additional receptors [39].

In terms of induction of immunity, it is difficult to generalise about DNA vaccines. Site and procedure of injection have critical influence on the immune activation. Muscle and skin cells are clearly able to act as antigen depots. The skin contains antigen presenting cells (APCs), hence capable of priming the immune system [40]. Roos *at al*. have optimised intra-dermal EP mediated PSA DNA vaccination and effectively induced PSA-specific T cells [41]. However, after i.m. plasmid delivery, it is likely that cross-presentation to APCs is the major route to priming [42]. The uncertainty on this point makes rational design more difficult. A recent investigation of the route of access of exogenous phagosomes to the MHC class I pathway could have relevance. The phagosomes apparently carry elements of the endoplasmic reticulum, creating organelles capable of antigen processing for induction of cytotoxic T cell responses [43,44]. It is conceivable that transfected depot cells undergoing apoptosis can behave similarly. The process that conveys antigens to the APCs seems highly efficient in that DNA vaccines that produce only very low levels of antigen can induce all arms of the immune response [45]. However, there may be different requirements for priming or boosting immunity and to activate anti tumour immunity; both processes need to be efficient. It is also essential that tumour cells alone can boost the vaccine-induced response so that continuing pressure is maintained against emergent cells. Translation of the immune therapies to clinical practice requires important optimisation. Various regimens of vaccine base therapies have been reported previously [21,46]. However, on review of the literature it is still not established which vaccination schedule is superior. We have shown that repeated vaccination provided optimal immunological tumour protective effects in our setting. Furthermore, repeated EP driven vaccination was safe as all immunised mice remained healthy and no adverse effect or treatment related death was observed.

The effective delivery of the vaccine vector to the host cells is a prime step for achieving immune activation. We used selected parameters of EP as a tool to boost the transfection of the muscle cells [27]. The transfer of DNA into the cells is a process where the cells membranes are initially permeabilised and then the DNA moved by electrophoretic forces into cytosol during the following pulses. Because of this, it has been shown that small molecules can diffuse into permeabilised cells in the minutes before membrane resealing. In contrast, there is no gene transfer if the DNA is added after the pulse [47]. Electric pulse parameters optimal for plasmid delivery (in the region of 1200 V/cm, 6-8 pulses) are known to increase gene expression 100-fold in muscle and other tissues, and have been shown to enhance humoral immune responses [22]. The high voltage pulse was to induce electroporation in the cell membrane and the ensuing small voltage pulses were to create an electrophoretic field to assist movement of the negative charged DNA plasmid across the cells [48,49]. The adjuvant effects of low voltage pulses might consist of increased activation and migration of the APCs, higher transfection of relevant APCs, or increased cellular infiltration. The optimal conditions for DNA vaccination, therefore, depend on the capacity of electroporation to enhance cellular immunity, especially for cancer vaccines for which IFNγ producing CD8^+ ^T cells are critical. The requirements might be different for the induction of humoral immune responses, for which the induced gene expression level might be of greater importance. Muscle is the most commonly targeted tissue for vaccine delivery where gene expression may last in excess of six months. The dominant mechanism for priming of CD8^+ ^T cells by APCs, after DNA vaccination, is still a matter of debate and may vary depending on if DNA is delivered into the muscle or the skin.

Despite these promising effects, the clinical efficiency of the different immunotherapeutic strategies for the majority of patients with advanced prostate cancer is still limited owing to various immune evasion mechanisms mediated by tumours. One of the major challenges in developing tumour vaccines relates to the fact that as tumours grow, the immune system looses the ability to target tumour cells, because of development of several immune evading strategies. These mechanisms include down-regulation of different components of the MHC class I processing and presentation machinery, generation of antigen loss variants, production of inhibitory cytokines such as transforming growth factor-ß and IL10, and expression of apoptosis-inducing molecules [50,51]. DNA vaccines, such as phPSA, have potential to activate specific tumour protective immunity and have potential to overcome these tumour escape mechanisms. Hormone therapy and radiotherapy for prostate cancer can have stimulatory effects on immune system [52,53]. Hence, a DNA vaccine such as phPSA has potential to be used in conjunction with available treatment for prostate cancer. The translation of this vaccination in clinical trials is further supported by the work of Ottensmeier *et al*. [54]. They have shown that EP is a potent method for stimulating humoral responses induced by DNA vaccination (encoding PSMA) in prostate cancer patients. It is hoped that prostate tumour vaccines would be able to destroy tumour cells that have survived hormone-blockade or radiotherapy.

## Conclusions

In summary, we have evaluated a plasmid DNA vaccine and shown that this phPSA vaccine can generate effective, durable tumour specific immune responses. The four-dose regimen provided optimal tumour protection and this was further enhanced by co-administration of synthetic oligo CpG. Additionally, this *in vivo *EP mediated vaccination is a safe and effective modality for the treatment of prostate cancer and has potential to be use as a neo-adjuvant or adjuvant therapy.

## Competing interests

The authors declare that they have no competing interests.

## Authors' contributions

SA carried out mice experiments, performed the statistical analysis, interpreted the data, and drafted the manuscript. GC carried out the *in vitro *experiments and performed statistical analysis. MT and PS participated in design of the study, helped with data interpretation, and drafting the manuscript. GCOS and MT conceived the study, participated in its design, and coordinated and drafted the manuscript. All authors read and approved the final manuscript.
